# Bandgap energy modeling of the deformed ternary GaAs_1-u_N_u_ by artificial neural networks

**DOI:** 10.1016/j.heliyon.2022.e10212

**Published:** 2022-08-13

**Authors:** A. Tarbi, T. Chtouki, Y. Elkouari, H. Erguig, A. Migalska-Zalas, A. Aissat

**Affiliations:** a Laboratory of Condensed Matter and Renewable Energy, Faculty of Sciences and Technology, University Hassan II of Casablanca, BP146 Mohammedia, Morocco; b Superior School of Technology, Materials Physics and Subatomic Laboratory, Ibn-Tofail University, PB 242, 14000 Kenitra, Morocco; c Faculty of Science and Technology, Jan Dlugosz University in Czestochowa, Al. Armii Krajowej 13/15, 42201 Czestochowa, Poland; d Faculty of Technology, University of Saad Dahlab Blida. 1, Blida, Algeria

**Keywords:** Materials, N-III-V semiconductor, Bandgap energy, Optoelectronic, Sensors

## Abstract

Appraising the bandgap energy of materials is a major issue in the field of band engineering. To better understand the behavior of GaAs_1-u_N_u_ material, it is necessary to improve the applied calculation methodologies. The band anticrossing model (BAC) allows modeling of the bandgap energy when diluted nitrogen is incorporated into the material. The model can be improved using artificial neural networks (ANN) as an alternative solution, which is rarely applied. Our goal is to study the efficiency of the (ANN) method to gauge the bandgap energy of the material from experimental measurements, considering the extensive strain due to the lattice mismatch between the substrate and the material. This makes the GaAsN material controllable with (ANN) method, and is a potential candidate for the fabrication of ultrafast optical sensors.

## 1 Introduction

N-III-V semiconductors are promising materials in the field of optical communications, and are key devices for keeping the bandgap and laser emission wavelength insensitive to the temperature effect [1]. The difference in size between isovalent atoms of ns^2^np^3^ electron configuration results in an unusual and abrupt discontinuous change in the band structure. Ternary GaAs_1-u_N_u_ compounds contain incompatible chemicals [2], and are strong candidates for the design of photovoltaic cells. It is characterized by its ability to adjust its energy band, which increases the absorption range of the solar spectrum with insensitivity to temperature.

The small size of nitrogen compared to arsenic causes tensile strain in the lattice and a large spin-orbit division [3, 4]. To explore the potential of this ternary compound, many reports have shown that nitrogen considerably narrows the bandgap energy, shifting the conduction band downwards [5], and adding 0%–5% nitrogen in GaAs shifts the bandgap energy at a rate of approximately 180 meV per 1% nitrogen [6, 7, 8].

Several models have been used to describe the physical mechanism responsible for this reduction, including have been used pseudopotential model [9], local density approximation LDA [10], combination of LDA and empirical pseudopotential method [11].

The BAC model [12, 13, 14] was developed in order to characterize the unusual behavior of the bandgap energy of the so-called strongly mismatched alloy HMAs. It is applied when incorporating the anionic sub-lattice of a III-V or II-VI semiconductor with highly electronegative isoelectronic impurity atoms [15, 16].

The high electronegativity of nitrogen attracts electrons and creates fault states near the conduction band edge of the host material [17]. In addition to the large curvature of the bandgap energy, other characteristics were noticed when adding nitrogen such as an increase in the effective mass of electrons [18] and the weak influence of pressure on the bandgap energy [19]. The excess nitrogen concentration created numerous radiative aggregates in the active layer. This also quickly deteriorates the quality of the alloy. Furthermore, it shortens the diffusion length, weakens the photoluminescence efficiency, and increases the laser threshold current [20, 21, 22].

Owing to the incompatibility of the GaAs and GaN lattice parameters of approximately 20% and the electronegativity of the network, a deformation of the electrostatic potential occurs, which leads to the nonlinearity of the bandgap energy depending on the nitrogen content [8].

The artificial neural network method (ANN) has been used to solve several problems in different fields; it is a logic programming technique developed that is based on learning from experimental or analytical/theoretical data available to solve complex problems [23, 24, 25, 26], which maps the input parameters to a specific output, and the formal neuron or perceptron mimics the functioning of a biological neuron to perform functions such as learning, memorization and decision, which is the basis of an ANN method. In this study, we calculated the bandgap energy of GaAN using an ANN and we conducted a comparative study between the experimental results, the results obtained by the ANN method, and the conduction band anticrossing model (CBAC) to test the robustness of the ANN method. After validating our ANN model, we simulated the effect of lattice mismatch on the bandgap.

## 2 Theoretical approach

The coupling between the localized states associated with the N atom of energy E_N_ = 1.65 eV, and the delocalized states of the minimum Γ of the conduction band of the host material is described by the BAC model [27], its expression is given by Eq. (1):

(1) $${\text{E}}_{\text{g}}^{\pm }\left(\text{u}\right)=0.5\left|{\text{E}}_{\text{N}}+{\text{E}}_{\text{c}\left(\text{GaAs}\right)}\pm \sqrt{{\left({\text{E}}_{\text{N}}-{\text{E}}_{\text{c}\left(\text{GaAs}\right)}\right)}^{2}+4{\text{V}}_{\text{MN}}{\left(\text{u}\right)}^{2}}\right|$$

V_MN_ expresses the interaction between energy states, it is given by the following Eq. (2):

(2) $${\text{V}}_{\text{MN}}=2.7\sqrt{\text{u}}$$

**Figure 1:**
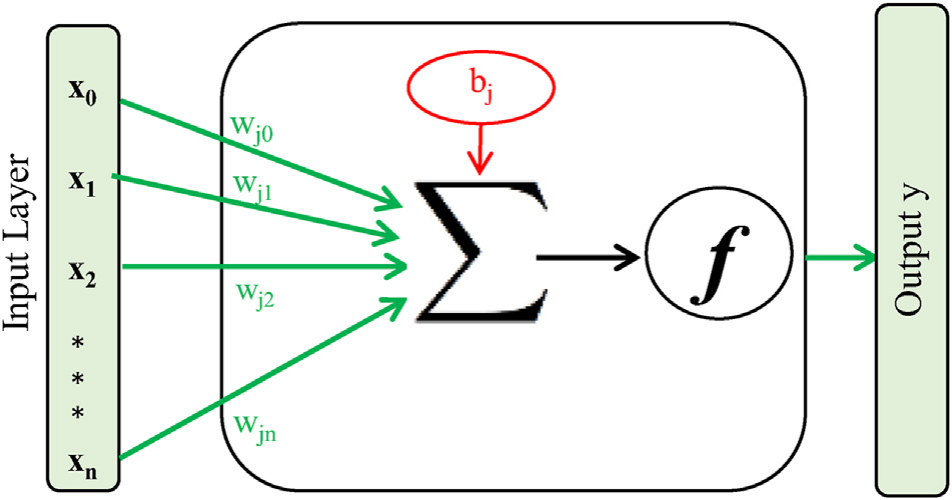
Methodology for information processing using neurons.

On the other hand, the ANN method is a mathematical tool used for modeling and prediction [28]. The function of a trained formal neuron is to weigh the input values as shown in Figure 1 and process the information by summing the bias-weighted inputs [29]. It then passes the results to an activation function [30]. The output is given by the following Eq. (3) [31, 32]:

(3) $$\text{y}=\text{f}\left(\overset{\text{n}}{\underset{\text{i}=1}{\sum }}\left({\text{w}}_{\text{ji}}{\text{x}}_{\text{ji}}\right)+{\text{b}}_{\text{j}}\right)$$

where:

y is the output of the current node of the hidden layer j,
n is the number of entries of the current layer,
x_i_ is the entry of the current hidden layer of the previous layer i,
w_ji_ is the weight that connects the current and the previous layers,
b_j_ is the bias, and f is the activation function.

In this study, we used the multilayer perceptron MLP, which consists of several disjoint layers connected to propagation (feedforward), while neurons of the same layer do not communicate with each other. The adjustment of weights and biases summarizes the digital knowledge of the network.

The learning of the direct reaction network is performed by an algorithm for optimizing the error function, and we chose the Levenberg-Marquardt (LM) backpropagation algorithm, which is characterized by high precision and fast quadratic convergence, approaching the minimum in a linear manner [33, 34]. The variance indicators are given by Eqs. (4) and (5):

(4) $$\text{RMSE}={\left(\frac{1}{\text{n}}\sum_{\text{i}=1}^{\text{n}}{\left({\text{E}}_{{\text{g}}_{\text{pred}}}-{\text{ ​E}}_{{\text{g}}_{\text{exp}}}\right)}^{2}\right)}^{1/2}$$

(5) $${\text{R}}^{2}=1-\left(\frac{\sum_{\text{i}=1}^{\text{n}}{\left({\text{E}}_{{\text{g}}_{\text{pred}}}-{\text{ ​E}}_{{\text{g}}_{\text{exp}}}\right)}^{2}}{\sum_{\text{i}=1}^{\text{n}}{\left({\text{E}}_{{\text{g}}_{\text{av},\text{exp}}}-{\text{ ​E}}_{{\text{g}}_{\text{exp}}}\right)}^{2}}\right)$$

where.

RMSE is the root-mean-square error,
${\text{R}}^{2}$ is the R-squared
E_gpred_ is the bandgap energy predicted by ANN model,
E_g exp_ is the experimental bandgap energy,
E_g av exp_ is the average experimental bandgap energy.

are used to evaluate of the quality of the prediction [35, 36]. The lattice mismatch creates a perpendicular strain ${\text{ε}}_{⊥}$ to the plane of the surface and another parallel strain, which are expressed by the following Eqs. (6) and (7) [37]:

(6) $$\varepsilon_{⊥}\left(\text{u}\right)=-2.\frac{C_{12}\left(\text{u}\right)}{C_{11}\left(\text{u}\right)}\varepsilon_{∥}\left(\text{u}\right)$$

(7) $$\varepsilon_{∥}\left(\text{u}\right)=\frac{\text{Δ}a\left(\text{u}\right)}{a_{s}}$$

where:

Δa is the difference between the lattice parameters of the substrate and epitaxial layer.
C_ij_ is the elastic constant GaAs_1-u_N_u_.

The bandgap energy of the compressed material is modeled by the following Eq. (8):

(8) $${\text{E}}_{\text{ghh}}\left(\text{u}\right)={\text{E}}_{\text{g}}\left(\text{u}\right)+\text{Δ}{\text{E}}_{\text{c}}^{\text{h}}\left(\text{u}\right)-\text{Δ}{\text{E}}_{\text{v},\text{av}}^{\text{h}}\left(\text{u}\right)-\text{Δ}{\text{E}}_{\text{hh}}^{\text{s}}\left(\text{u}\right)$$

In the case of a layer subjected to a tensile strain, the bandgap energy is given by the following Eq. (9):

(9) $${\text{E}}_{\text{glh}}\left(\text{u}\right)={\text{E}}_{\text{g}}\left(\text{u}\right)+\text{Δ}{\text{E}}_{\text{c}}^{\text{h}}\left(\text{u}\right)-\text{Δ}{\text{E}}_{\text{v},\text{av}}^{\text{h}}\left(\text{u}\right)-\text{Δ}{\text{E}}_{\text{lh}}^{\text{s}}\left(\text{u}\right)$$

With $\text{Δ}{\text{E}}_{\text{hh}}^{\text{s}}$ and $\text{Δ}{\text{E}}_{\text{lh}}^{\text{s}}$ translate the displacement of the valence band by the effect of the deformation due to shear [27]. $\text{Δ}{\text{E}}_{\text{v},\text{av}}^{\text{h}}$ and $\text{Δ}{\text{E}}_{\text{c}}^{\text{h}}$ represent the energy displacements of the valence band and conduction band due to hydrostatic deformation, respectively [38].

The Matthews - Blakeslee model [39] makes it possible to describe the critical thickness using the following Eq. (10):

(10) $$L_{c}=\frac{a_{s}}{2\sqrt{2}\pi \varepsilon \beta }\frac{1-0.25\sigma }{1-\sigma }\text{ln}\left(\frac{L_{c}\sqrt{2}}{a_{s}}+1\right)$$

σ represents the Poisson's ratio, which is given by the following expression (11):

(11) $$\sigma =\frac{C_{12\text{ ​sub}}}{C_{11\text{ ​sub}}+C_{12\text{ ​sub}}}$$

where:

β is a coefficient which depends on the structure of the material.
$C_{11\text{ ​sub}}$ and $C_{12\text{ ​sub}}$ are elastic constants of the GaAs substrate.

## 3 Results and discussion

**Figure 2:**
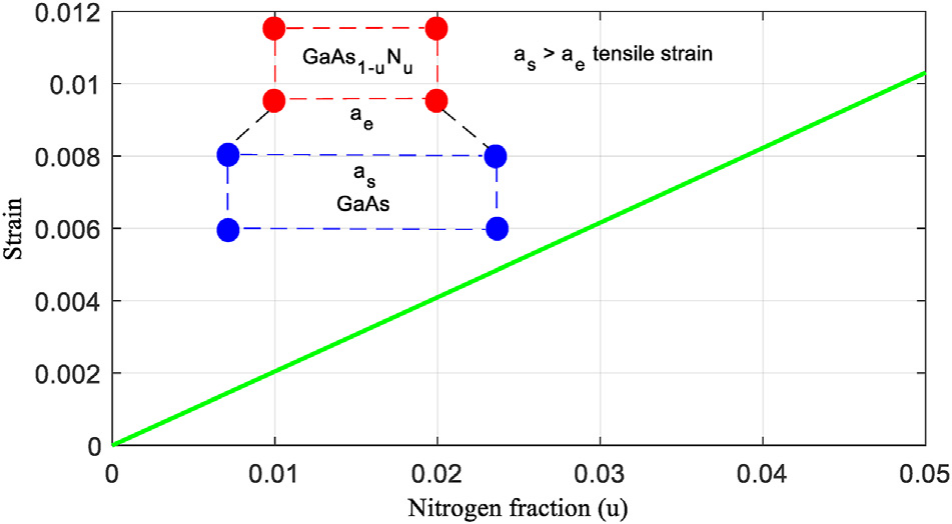
Extensive strain due to lattice mismatch between GaAs_1-u_N_u_ on GaAs substrate.

Figure 2 presents the strain as a function of the concentration N, it increases linearly with the rate of N, it is always positive, which shows that there is extensive deformation due to the lattice mismatch; therefore, the valence band nondegenerate closest to the conduction band is the energy band related to the light holes.

**Figure 3:**
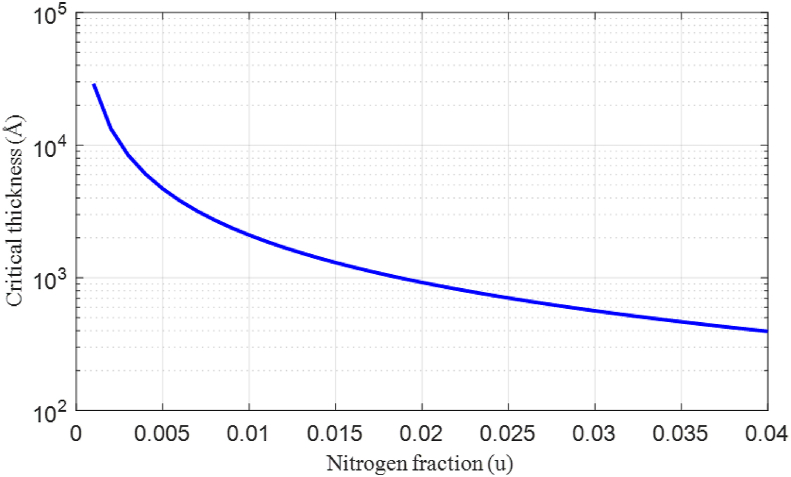
Variation in the critical thickness of GaAsN ternary material as a function of N concentration.

Figure 3 shows the variation in the bandgap energy of the sub-bands (${{\text{E}}_{\text{g ​}}^{-}}_{\text{BAC}}$ and ${{\text{E}}_{\text{g ​ ​}}^{+}}_{\text{BAC}}$) according to the concentration of N in the material GaAs_1-u_N_u_, as calculated by the BAC model. We notice that the increase in N creates repulsion between the two sub-bands and reflects a contradictory behavior between them: the energy level of the sub-band ${{\text{E}}_{\text{g ​ ​}}^{+}}_{\text{BAC}}$ increases and the other ${{\text{E}}_{\text{g ​}}^{-}}_{\text{BAC}}$ decreases.

In the absence of N, the value $\text{Δ}{\text{E}}_{\text{g}}$ is equal to 0.23 eV, however, when the concentration of N becomes important (4%), $\text{Δ}{\text{E}}_{\text{g}}$ reaches 1.10 eV. The energy of the sub-band ${{\text{E}}_{\text{g}}^{-}}_{\text{BAC}}$ decreases from 1.42 eV to 0.98 eV with a rate of decrease equal to 31% by increasing the concentration of N from 0% to 4%.

Figure 3 describes the critical thickness of the ternary GaAsN material as a function of the N content, which decreases monotonically with increasing strain owing to the addition of nitrogen. The material becomes unstable and can cause irreversible deformation at high nitrogen concentrations.

**Figure 4:**
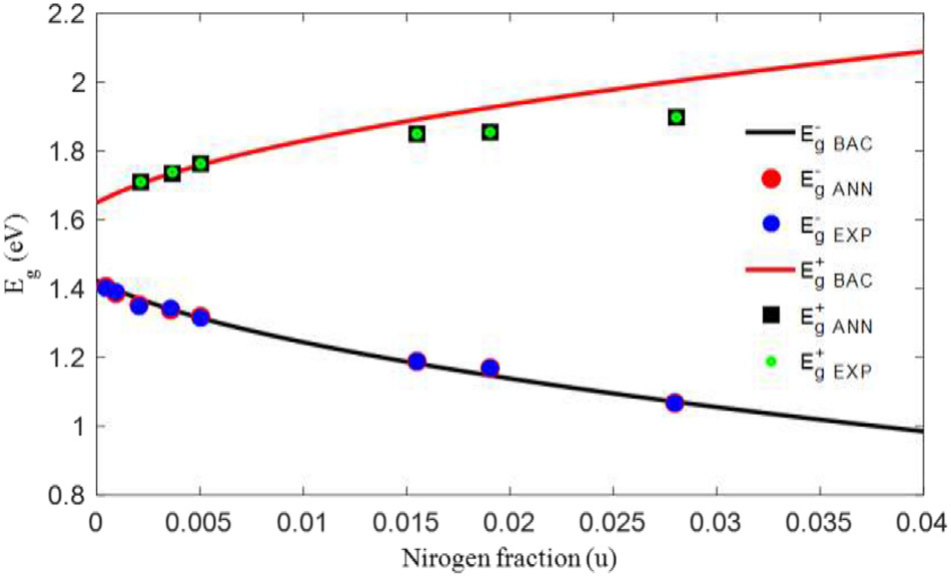
Comparison of the bandgap energy of the subbands calculated by the (BAC) model, estimated by the (ANN) model with the experimental data [40] at T = 300 K.

Figure 4 also makes it possible to compare the results obtained with the BAC and ANN models (${{\text{E}}_{\text{g ​}}^{-}}_{\text{ANN}}$ and ${{\text{E}}_{\text{g ​}}^{+}}_{\text{ANN}}$) with the experimental results (${{\text{E}}_{\text{g ​}}^{-}}_{\text{EXP}}$ and ${{\text{E}}_{\text{g ​}}^{+}}_{\text{EXP}}$) [40]. We used the TanSig function, a single hidden layer, and eight neurons to estimate ${{\text{E}}_{\text{g ​}}^{-}}_{\text{ANN}}$. The purelin function, two hidden layers with three and six neurons were used to estimate ${{\text{E}}_{\text{g ​}}^{+}}_{\text{ANN}}$. The R^2^ criterion in both cases is almost equal to 1, which confirmed that the results of the ANN model were in perfect agreement with the experimental data.

**Figure 5:**
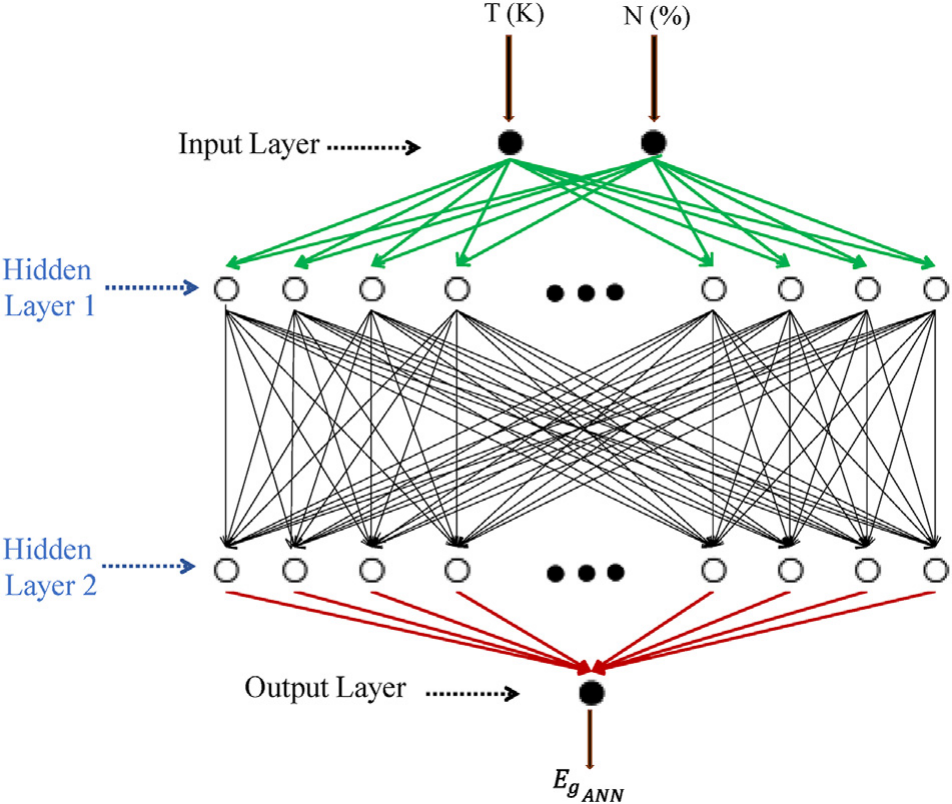
Topology of the multilayer Artificial Neural Network used for the prediction of ${{\text{E}}_{\text{g ​}}}_{\text{ANN}}$.

To predict the variation in the bandgap energy as a function of the N concentration, we used the ANN method optimized by the Levenberg-Marquardt algorithm. As shown in Figure 5, the architecture of the network includes an input layer composed of two input nodes (the concentration of N and the temperature), an output layer that estimates ${\text{E}}_{\text{g ​ANN}}$ and two hidden layers with 20 neurons in each layer. The optimal activation function was log-sigmoid and the optimal transfer function was purelin. 70% of the experimental data [1, 41] were used for training, 15% for testing and 15% for validation.

**Figure 6:**
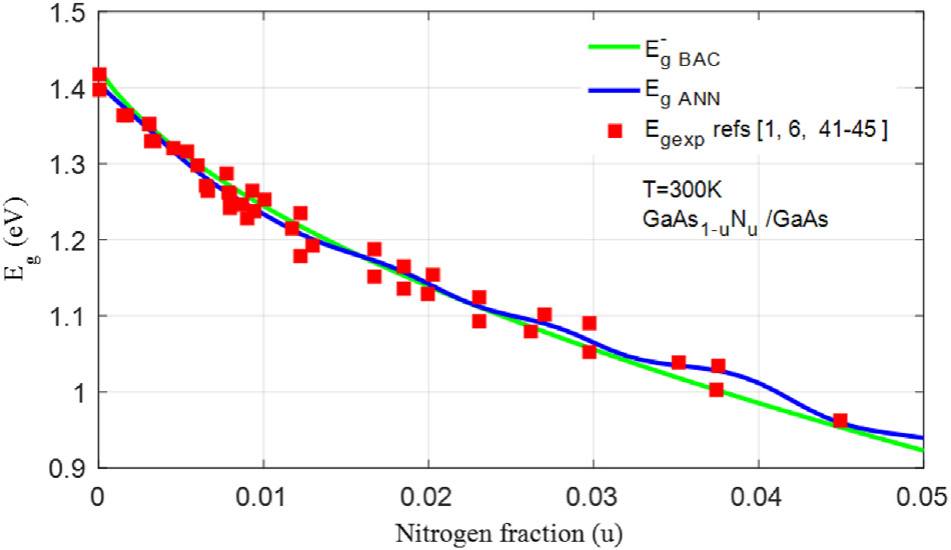
Representation of experimental bandgap energy [1, 6, 41, 42, 43, 44, 45], calculated by the BAC model, and estimated by ANN-LM model.

**Figure 7:**
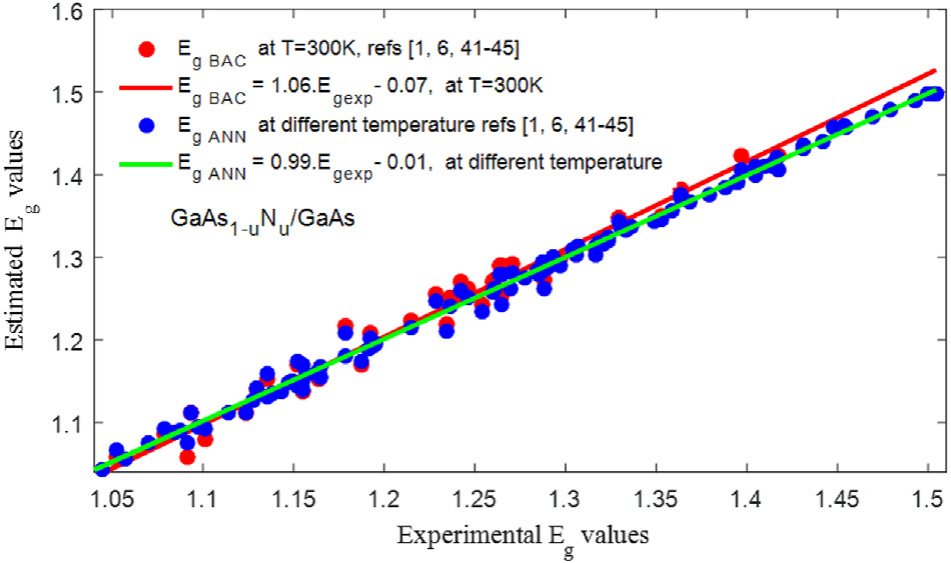
Correlation between the experimental values and the values estimated by ANN-LM model for a temperature between 0 K and 300 K, and between the experimental values and the values calculated by the BAC model ${{\text{E}}_{\text{g ​}}}_{\text{BAC}}$ at T = 300 K [1, 6, 41, 42, 43, 44, 45].

Figure 6 shows the variation in bandgap energy as a function of the N concentration. The bandgap energy ${{\text{E}}_{\text{g ​}}}_{\text{ANN}}$ matches with the experimental results. In order to validate our model, we calculated the correlation between the experimental values ${{\text{E}}_{\text{g ​}}}_{\text{exp}}$, the values ${{\text{E}}_{\text{g}}}_{\text{ANN}}$ estimated by ANN-LM, and the values ${{\text{E}}_{\text{g}}^{-}}_{\text{BAC}}$ calculated using the BAC model. As shown in Figure 7, the slope is closer to 1 for ${{\text{E}}_{\text{g ​}}}_{\text{ANN}}$, which shows that the values of ${{\text{E}}_{\text{g ​}}}_{\text{ANN}}$ are close to the experimental values, and the RMSE and the ${\text{R}}^{2}$ reached by ANN-LM, RMSE_ANN_ = 0.0095 and R^2^_ANN_ = 99.44% are better than those found using the BAC model RMSE_ANN_ = 0.0181 and R^2^_ANN_ = 97.31%.

**Figure 8:**
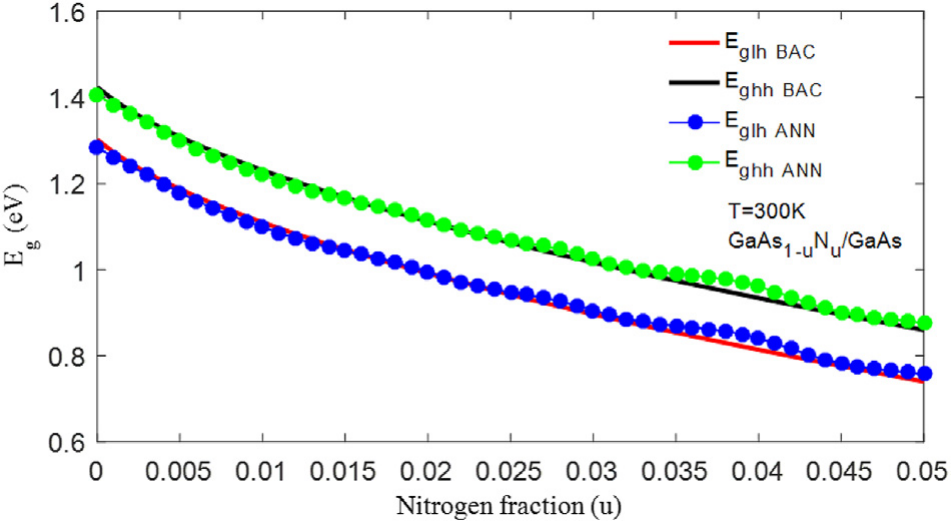
Representation of the bandgap energy ${\text{E}}_{\text{glh}}$ associated with light holes and ${\text{E}}_{\text{ghh}}$ associated with heavy holes, as a function of the N concentration, calculated by the BAC model, and estimated by ANN-LM model.

Figure 8 shows the bandgap energy of the material subjected to reversible strain calculated using the BAC model and estimated using the ANN-LM model. The energy ${{\text{E}}_{\text{glh ​}}}_{\text{ANN}}$ and ${{\text{E}}_{\text{ghh ​}}}_{\text{ANN}}$ estimated by the ANN model decrease with fluctuation, which shows that the physical process has been modeled by a complex equation of high nonlinearity, whereas the energy ${{\text{E}}_{\text{glh}}}_{\text{BAC}}$ and ${{\text{E}}_{\text{ghh}}}_{\text{BAC}}$ calculated by the BAC model decrease in a hyperbolic manner with very little curvature.

## 4 Conclusion

In this article, we report a comparative study of the bandgap energy of the material GaAs_1-u_N_u_/GaAs calculated using the BAC model and predicted by the ANN method. We found that the ANN model gave values very close to the experimental values, and its performance was ensured by calculating the error indicators. The precision determined by the RMSE_ANN_ = 0.005 and R^2^_ANN_ = 99.44% of the ANN model was better than that of the BAC model, RMSE_BAC_ = 0.0181 and R^2^_BAC_ = 97.31%, which means that the improvement in R^2^ and RMSE reaches ΔR^2^ = 2.14% and ΔRMSE = 0.0131, respectively. The deformation of the material due to lattice mismatch leads to lifting of the degeneration of the valence band. When gradually incorporating nitrogen, the energy levels ${\text{E}}_{\text{ghh}}$ and ${\text{E}}_{\text{glh}}$ calculated by the BAC model decreased in a hyperbolic manner, whereas those predicted by the ANN model decreased with fluctuation. These results demonstrate the ability of the ANN model to describe bandgap energy with a complex function and high nonlinearity. In future work, we will use the ANN method for N-III-V quaternary and quinary structures in photovoltaic and optoelectronic applications.

## Declarations

### Author contribution statement

A. Tarbi, T. Chtouki, H. Erguig, A. Migalska-Zalas, A. Aissat: Conceived and designed the experiments; Performed the experiments; Analyzed and interpreted the data; Contributed reagents, materials, analysis tools or data; Wrote the paper.

### Funding statement

This research did not receive any specific grant from funding agencies in the public, commercial, or not-for-profit sectors.

### Data availability statement

Data included in article/supplementary material/referenced in article.

### Declaration of interests statement

The authors declare no conflict of interest.

### Additional information

No additional information is available for this paper.
